# Semen Modulates the Expression of NGF, ABHD2, VCAN, and CTEN in the Reproductive Tract of Female Rabbits

**DOI:** 10.3390/genes11070758

**Published:** 2020-07-07

**Authors:** Jaume Gardela, Amaia Jauregi-Miguel, Cristina A. Martinez, Heriberto Rodriguez-Martinez, Manel Lopez-Bejar, Manuel Alvarez-Rodriguez

**Affiliations:** 1 Division of Children’s and Women Health (BKH), Obstetrics and Gynecology, Department of Biomedical and Clinical Sciences (BKV), Linköping University, 58185 Linköping, Sweden; jaume.gardela@uab.cat (J.G.); cristina.martinez-serrano@liu.se (C.A.M.); heriberto.rodriguez-martinez@liu.se (H.R.-M.); 2 Department of Animal Health and Anatomy, Veterinary Faculty, Universitat Autònoma de Barcelona, 08193 Bellaterra, Spain; manel.lopez.bejar@uab.cat or; 3 Division of Molecular Medicine and Virology (MMV), Linköping University, 58185 Linköping, Sweden; amaya.jauregi.miguel@liu.se; 4 College of Veterinary Medicine, Western University of Health Sciences, Pomona, CA 91766, USA

**Keywords:** gene expression, qPCR, female reproductive tract, induced ovulator

## Abstract

Semen changes the gene expression in endometrial and oviductal tissues modulating important processes for reproduction. We tested the hypothesis that mating and/or sperm-free seminal plasma deposition in the reproductive tract affect the expression of genes associated with sperm-lining epithelium interactions, ovulation, and pre-implantation effects (nerve growth factor, NGF; α/β hydrolase domain-containing protein 2, ABHD2; C-terminal tensin-like protein, CTEN or TNS4; and versican, VCAN) in the period 10–72 h post-mating. In Experiment 1, does (*n* = 9) were treated with gonadotropin-releasing hormone (GnRH) (control), GnRH-stimulated, and vaginally infused with sperm-free seminal plasma (SP-AI), or GnRH-stimulated and naturally mated (NM). In Experiment 2, does (*n* = 15) were GnRH-stimulated and naturally mated. Samples were retrieved from the internal reproductive tracts (cervix-to-infundibulum) 20 h post-treatment (Experiment 1) or sequentially collected at 10, 24, 36, 68, or 72 h post-mating (Experiment 2, 3 does/period). All samples were processed for gene expression analysis by quantitative PCR. Data showed an upregulation of endometrial *CTEN* and *NGF* by NM, but not by SP-AI. The findings suggest that the *NGF* gene affects the reproductive tract of the doe during ovulation and beyond, influencing the maternal environment during early embryonic development.

## 1. Introduction

Rabbits (*Oryctolagus cuniculus*) are considered to be game, vermin, laboratory animals, pets, or livestock, and are principally consumed in Mediterranean Europe [1]. Unlike many other livestock species, rabbits are induced ovulators, requiring the generation of genital-somatosensory signals during coitus to activate midbrain and brainstem noradrenergic neurons and generate the preovulatory peak of gonadotropin-releasing hormone (GnRH) [2,3]. This mating-induced output of GnRH, which is of a far greater magnitude than what has been reported for other species, causes an immediate release of luteinizing hormone from the anterior pituitary that results in ovulation [2].

Ejaculate deposition during mating affects the molecular and cellular functions near and distant to the insemination site [4]. In rabbits, approximately 1 mL of ejaculate volume containing about 300 million spermatozoa suspended in seminal plasma (SP) is deposited vaginally after mating [5]. When spermatozoa reach the oviduct, within minutes in rabbits [6], only a small number of them attaches to the oviductal epithelium [7,8]. Sperm transport along the female reproductive tract follows two phases. The first is a rapid phase, where the myometrium and myosalpinx move a vanguard of spermatozoa to the upper ampulla and beyond, ensuring that a sperm group is cleared from the oviduct before ovulation [9]. The second is a sustained phase that colonizes the tubal sperm reservoir until ovulation takes place [10]. Previous research has proposed the distal isthmus (adjacent to, or perhaps part of, the utero-tubal junction) as the principal anatomical region restricting rabbit spermatozoa, acting as a sperm reservoir [10]. In these reservoirs, the spermatozoa are entrapped and safeguarded from the female immune system by the fluid and extracellular matrix present in the lumen of the oviduct [11].

Two of the principal components of the endometrial extracellular matrix are versican and hyaluronic acid [12]. Both may interact with specific binding domains, creating open networks to facilitate cell division, movement, and sorting [13]. The CD44, a cell-surface glycoprotein involved in cell-cell adhesion, is the best-characterized ligand to hyaluronic acid that also binds to versican [14]. It is present in a wide variety of cells and tissues, including spermatozoa, facilitating the adhesion and protection of those cells in the oviductal sperm reservoir [11].

Shortly after ovulation, spermatozoa are gradually released from the sperm reservoir and moved to the ampullar site of fertilization [8,10]. Modifications of the sperm and/or the epithelium lining surface proteins produced during sperm capacitation may reduce the sperm binding affinity to the oviductal epithelium [8]. This, together with the attainment of hyperactivation motility, which is required to facilitate sperm-oocyte contact and penetration of the zona pellucida [15], would result in the detachment of spermatozoa from the oviductal sperm reservoir. Ovulation is induced around 10 h after coitus or GnRH stimulation [16], and fertilization occurs 2–3 h after ovulation [15]. All of these events are the result of endocrine and neuronal interplay mainly connected to mating, but perhaps in concerted relation to the entry of semen and their effects on the genomic response by the female genital tract, as previously suggested in other species [17,18,19].

The nerve growth factor (NGF) not only participates in the differentiation, plasticity, and phenotype of sensory and sympathetic neurons [20], but has also been proposed as a key ovulation-inducing factor in rabbits and other induced ovulators, due to its presence in the SP [3,21,22,23,24]. In the genital tract, NGF may display different effects, from activating sperm motility via its cytoskeleton influence, as it occurs during neuronal growth, to preventing embryo rejection through the inhibition of local immune responses [20].

The enzyme α/β hydrolase domain-containing protein 2 (ABHD2) is involved in the control of sperm hyperactivation via progesterone and cation channels of sperm, also known as CatSper Ca^2+^ channels [25,26,27]. Versican (encoded by *VCAN*), an extracellular matrix component [12], plays a fundamental role in embryo implantation [28]. Recently, differential *VCAN* and *ABHD2* expression have been reported in the porcine endometrium after mating and sperm-free SP infusion [29]. Besides, differential expression of the gene encoding for the C-terminal tensin-like protein (CTEN; also known as tensin 4, TNS4), a member of the tensin family crucial for cell-matrix adhesion [30], has been reported in the porcine endometrium triggered by spermatozoa and sperm-free SP [19]. Altogether, these genes may not only participate in the endometrium-sperm interaction, but also in the interaction between the genital environment and the zygotes, which is a matter that has not yet been explored in induced ovulators.

The present study tested the effect of mating and/or seminal plasma on the gene expression of the tubular internal reproductive tract of does, with special reference to genes specifically associated with sperm-lining epithelium interactions (adhesion), ovulation, and pre-implantation effects (which can be separated from the free embryo influence) in the period 10–72 h post-mating, during which all these events occur.

## 2. Materials and Methods

### 2.1. Ethics Statement

Rabbits were handled according to the principles of animal care published by Spanish Royal Decree 1201/2005 (BOE, 2005: 252:34367-91) and the Directive 2010/63/EU of the European Parliament and the Council of 22 September 2010 on the protection of animals used for scientific purposes (2010; 276:33-79). The Committee of Ethics and Animal Welfare of the Universitat Autónoma de Barcelona, Spain, approved this study (Expedient #517).

### 2.2. Animals

Six New Zealand White (NZW) adult rabbit bucks and 24 NZW adult rabbit does (from seven to thirteen months old) from the nucleus colony at the farm of the Institut de Recerca i Tecnologia Agroalimentaries (IRTA-Torre Marimon, Caldes de Montbui, Barcelona, Spain) were used in this study. Each animal was housed in a single cage (85 × 40 × 30 cm) equipped with plastic footrests, a feeder (restricted to 180 g/day of an all-mash pellet), and nipple-drinkers (ad libitum access to water). Animals were kept under a controlled photoperiod of 16 h of light and 8 h of darkness, and a range of temperature between 15 and 20 °C in winter and 20 and 26 °C in summer, with a relative humidity of 60% to 75% maintained by a forced ventilation system.

All males started to be trained with an artificial vagina at 4.5 months of age. A homemade polyvinyl chloride artificial vagina containing water at a temperature of 50 °C was used. One ejaculate was collected per male. Ejaculates that contained urine and calcium carbonate deposits on visual inspection were discarded.

### 2.3. Experimental Design

**Figure 1:**
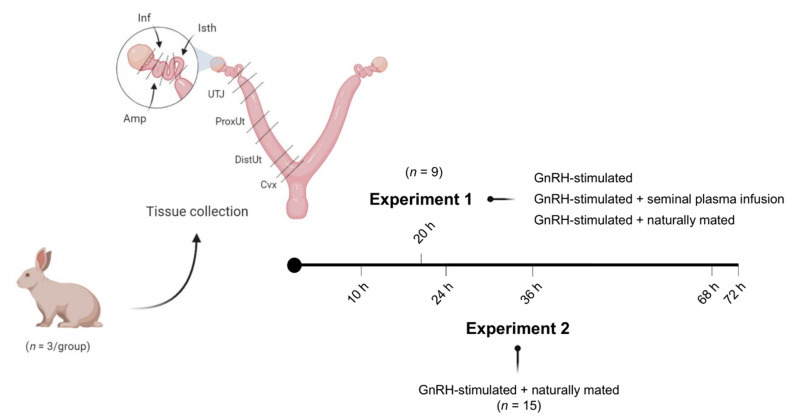
Representation of the experimental design and tissue sections obtained from does. Sequential tissue segments derived from does: endocervix (Cvx), distal uterus (DistUt), proximal uterus (ProxUt), utero-tubal junction (UTJ), isthmus (Isth), ampulla (Amp), and infundibulum (Inf). Intramuscular injection of 0.03 mg gonadotropin-releasing hormone (GnRH) was used to induce ovulation in all groups of both experiments.

Figure 1 displays the experimental design followed in this study. Two separate experiments were performed.

In Experiment 1 (*n* = 9), gene expression analyses for *ABHD2*, *NGF*, *CTEN*, and *VCAN* were performed in sequential segments of the female reproductive tracts randomly chosen from one lateral side. Tissue samples were collected 20 h post-induction of ovulation with 0.03 mg gonadotropin-releasing hormone (GnRH; Fertagyl^®^, Esteve Veterinaria, Barcelona, Spain) intramuscularly (im) (control of ovulation; Control, *n* = 3), 20 h post-induction of ovulation with 0.03 mg GnRH im and sperm-free SP vaginal infusion (SP-AI, *n* = 3), and 20 h post-induction of ovulation with 0.03 mg GnRH im and natural mating (NM, *n* = 3). The control group was established to highlight the effects of the SP infusion and mating, thus counteracting ovulation effects.

In Experiment 2, 15 does were sequentially euthanized at 10, 24, 36, 68, or 72 h post-induction of ovulation with 0.03 mg GnRH im and natural mating (*n* = 3/collection time). Reproductive tract tissues, as described in Experiment 1, were collected for *ABHD2*, *NGF*, *CTEN*, and *VCAN* gene expression analysis. The 10 h post-mating group was established as the reference group.

### 2.4. Mating and Semen Collection

The does included in the mating group of Experiment 1 and 2 were sequentially mated with two randomly selected bucks to diminish male-variation effects after the hormonal induction of ovulation with 0.03 mg GnRH im. The ovulation was assumed to happen at 10 h after GnRH stimulation for all groups. Additionally, semen was collected from the same rabbit bucks through an artificial vagina, as described above. The sperm-free SP was obtained after centrifugation at 2000× *g* for 10 min and checked for the absence of spermatozoa. The harvested sperm-free SP was immediately used as a pool for sperm-free SP vaginal infusions of Experiment 1 after the hormonal induction of ovulation with 0.03 mg GnRH im.

### 2.5. Tissue Sample Collection

For each experimental condition, the does were euthanized by the administration of 600 mg pentobarbital sodium (Dolethal, Vetoquinol, Madrid, Spain) intravenously (marginal ear vein). Immediately thereafter, the right female reproductive tracts were chosen from the right lateral side in all animals and retrieved and segmented in seven consecutive compartments (endocervix, distal uterus, proximal uterus, utero-tubal junction, distal isthmus, ampulla, and infundibulum; Figure 1). In Experiment 2, before segmentation of the internal genital tract, the entire oviduct was isolated from the uterus and the ovary by cutting below the utero-tubal junction. Embryos were collected by flushing the oviduct through the infundibulum (phosphate buffer saline supplemented with 5% fetal calf serum and 1% antibiotic-antimycotic solution). All genital segments were stored in RNAlater solution at −80 °C.

### 2.6. Quantitative PCR Analyses

Total RNA was extracted following a TRIzol-based protocol. Tissues were mechanically disrupted in a 2-mL tube with 1 mL TRIzol using a bead mill (29 beats/s, 2 min) (TissueLyser II with 7 mm stainless steel beads, Qiagen, Sollentuna, Sweden). The homogenized tissues were centrifuged at 12,000× *g* for 10 min at 4 °C. The supernatants were transferred to a new 2-mL tube and mixed by hand with bromochloropropane (100 µL/mL homogenized). After 5-min of incubation at room temperature, the mixtures were centrifuged 12,000× *g* for 15 min at 4 °C. The aqueous phases obtained were transferred to a new 1.5-mL tube with isopropanol and RNA precipitation solution (1.2 M NaCl and 0.8 M Na_2_C_6_H_6_O_7_) (250 µL of each/500 µL aqueous phase). After 10-min of incubation at room temperature, the mixtures were centrifuged 12,000× *g* 10 min at 4 °C. The supernatants were discarded, and 1 mL of 75% ethanol was added to each tube. The tubes were centrifuged 7500× *g* for 5 min at 4 °C and the supernatants were discarded. The RNA pellets obtained were dried for 30 min in the fume hood before their dissolution in 30 µL of RNase free water for 30 min on ice. The RNA concentration of the extracts was determined from the absorbance of 260 nm with Thermo Scientific NanoDrop^TM^ 2000 (Fisher Scientific, Gothenburg, Sweden). All of the samples had a 260/280 nm absorbance ratio of 1.8–2.2. Additionally, the quality of the RNA was determined by the Agilent 2100 Bioanalyzer (Agilent Technologies, Palo Alto, CA, USA), using the samples with a RNA integrity number (RIN) value higher than 8. The synthesis of the first-strand cDNA was performed using the High-Capacity RNA-to-cDNA™ Kit (Applied Biosystems™, CA, USA), which consisted of 4 µg RNA in a final volume of 20 µL. After the synthesis, the samples were stored at −20 °C until further analyses.

A Quantitative Polymerase Chain Reaction (qPCR) was performed using the Real-Time PCR Detection System (CFX96™; Bio-Rad Laboratories, Inc; CA, USA). The reactions consisted of 2 µL of synthesized cDNA, 250 nM of forward and reverse specific gene primers, 5 µL of PowerUp™ SYBR™ Green Master Mix (Applied Biosystems™, CA, USA), and water to a final volume of 10 µL. The protocol was as follows: one cycle of uracil-DNA glycosylase (UDG) activation at 50 °C for 2 min; one cycle of denaturation at 95 °C for 2 min; and 40 cycles of denaturation at 95 °C for 5 s, annealing/extension at 60.2 °C for 30 s, and a melting curve at 60–95 °C (0.5 °C increments) for 5 s/step. Two technical replicates were used for each sample. The efficiencies of the primers were calculated using five different concentrations of the same cDNA sample (serial dilutions of 1/5) and the same protocol described above. Three technical replicates were used for each concentration. The gene relative expression levels were quantified using the Pfaffl method [31]. Two housekeeping genes were initially used for cDNA normalization (*β-ACTIN* and *GADPH*). After a preliminary analysis of the results, only *β-ACTIN* was constantly expressed through the tissues and treatments, and was chosen for further analyses. The primer sequences, product sizes, and efficiencies are shown in Table 1. For the *β-ACTIN* gene, commercial gene-specific PCR primers for rabbit samples were used (PrimePCR™ SYBR^®^ Green Assay: ACTB, Rabbit; Bio-Rad Laboratories, Inc; CA, USA). The amplicons of the qPCR were loaded into an agarose gel after being mixed with GelRed^®^ Nucleic Acid Gel Stain (Biotium, CA, USA) to confirm the product sizes. After running the test, the gel was imaged by a gel imaging system (ChemiDoc XRS+ System, BioRad Laboratories, Inc; CA, USA).

### 2.7. Statistical Analyses

All data were exported with CFX Maestro™ 1.1 software version 4.1.2433.1219 (Bio-Rad Laboratories, Inc; CA, USA). All data sets were analyzed for a normal distribution and homoscedasticity using the Shapiro–Wilk Normality test and Levene’s test. Log(x) transformation was used to restore a normal distribution prior to analysis. The statistical analysis was conducted in R version 3.6.1. [32] with *nlme* [33] to develop linear mixed-effects (LME) models and *multcomp* [34] to perform pairwise comparisons adjusted by Tukey’s test. Data are presented as the median [minimum, maximum], unless otherwise stated. The threshold for significance was set at *p* < 0.05.

Treatments of Experiment 1 (control, SP-AI, and NM) were included as fixed effects and the females as the random part of the model. Pairwise comparisons were adjusted by Tukey’s test.

A second LME model was used, including the different collection times of Experiment 2 (10, 24, 36, 68, and 72 h post-mating) as fixed effects and the females as the random part of the model. Post-hoc comparisons were performed using Tukey’s multiple comparisons test.

Additionally, the differential expression changes in the qPCR results among tissues (endocervix to infundibulum) in Experiments 1 and 2 were further re-analyzed, separately. Using the utero-tubal junction as an arbitrary anatomical compartment reference among all tissues examined, gene expression changes were compared, per gene, issued by the control, NM, or SP-AI (Experiment 1) or by different times post-mating (Experiment 2), among the anatomical regions of the female reproductive tract. The tissue was included as fixed effects and the females as the random part of the LME model. The LME model was followed by Tukey’s multiple comparison test, to analyze the differences among each anatomical region of the female reproductive tract. Data are presented as the median [minimum, maximum], unless otherwise stated. Data on the differential expression among tissues are presented as Row Z-Scores.

The first statistical analyses were conducted to determine gene expression changes among the experimental groups for each tissue section, whereas the second statistical analyses were conducted to determine the differences between the tissue segments for every experimental group. Both analyses used the same data (expression values), but were analyzed to emphasize, in the first analysis, the different treatments and post-mating times, and in the second analysis, the differential expression among tissues.

## 3. Results

### 3.1. Differential Gene Expression in the Rabbit Female Reproductive Tract 20 h after Natural Mating or Vaginal Infusion With Sperm-Free Seminal Plasma

**Figure 2:**
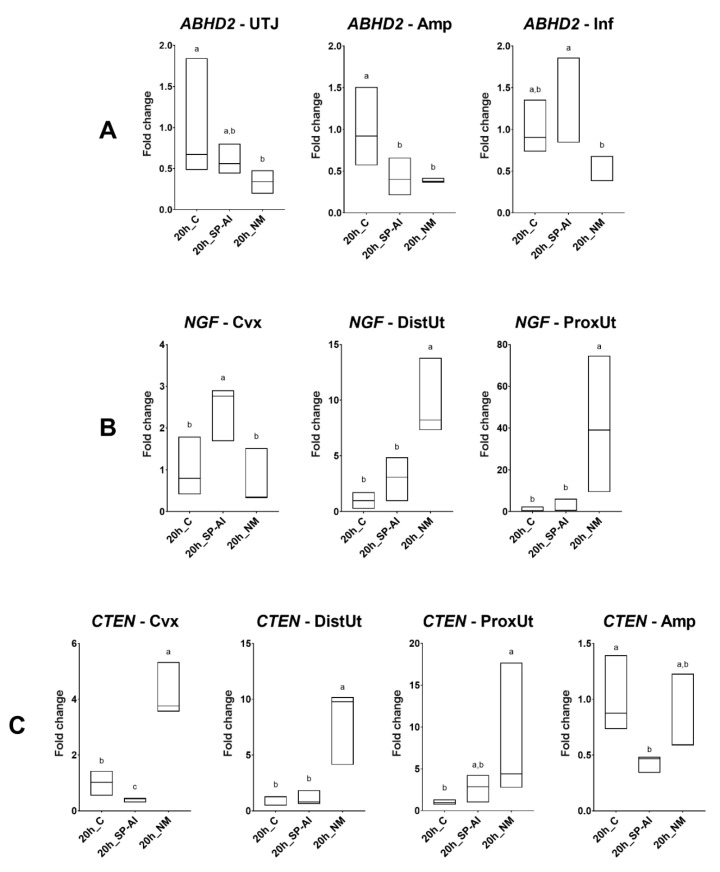
Results of Experiment 1. Gene expression of (**A**) *ABHD2*, (**B**) *NGF*, and (**C**) *CTEN* was statistically significant (*p* < 0.05) in the rabbit endocervix (Cvx), distal uterus (DistUt), proximal uterus (ProxUt), utero-tubal junction (UTJ), ampulla (Amp), and infundibulum (Inf) 20 h after the induction of ovulation with an intramuscular injection of 0.03 mg gonadotropin-releasing hormone (GnRH) (20 h_C, *n* = 3), 20 h post-GnRH-stimulation and seminal plasma vaginal infusion (20 h_SP-AI, *n* = 3), and 20 h post-GnRH-stimulation and natural mating (20 h_NM, *n* = 3). Fold changes relative to the control of the ovulation group are shown. Different letters (^a,b^) represent statistical differences between groups (*p* < 0.05). Median [minimum, maximum].

Differences in *ABHD2*, *NGF*, and *CTEN* expression among the groups included in Experiment 1 are displayed in Figure 2. No differences were found in the distal isthmus, which is the sperm reservoir established in the rabbit [10]. The *ABHD2* expression was downregulated by the NM group in the utero-tubal junction (*p* < 0.05), and downregulated by NM and SP-AI groups in the ampulla (*p* < 0.05). Conversely, the SP-AI group upregulated *ABHD2* expression in the infundibulum compared to the NM group (*p* < 0.01). The *NGF* expression was upregulated by the NM group in the distal and proximal uterus (*p* < 0.05). However, the SP-AI group upregulated *NGF* expression in the endocervix (*p* < 0.05). The *CTEN* expression was upregulated by the NM group in the endocervix, and proximal and distal uterus (*p* < 0.01). Additionally, the SP-AI group downregulated *CTEN* expression in the endocervix and ampulla (*p* < 0.01). The results obtained from all of the genes and tissues analyzed are depicted in Figure S1. 

### 3.2. Differential Gene Expression in the Rabbit Female Reproductive Tract from Ovulation (10 h Post-Mating) to up to 72 h Post-Mating

**Figure 3:**
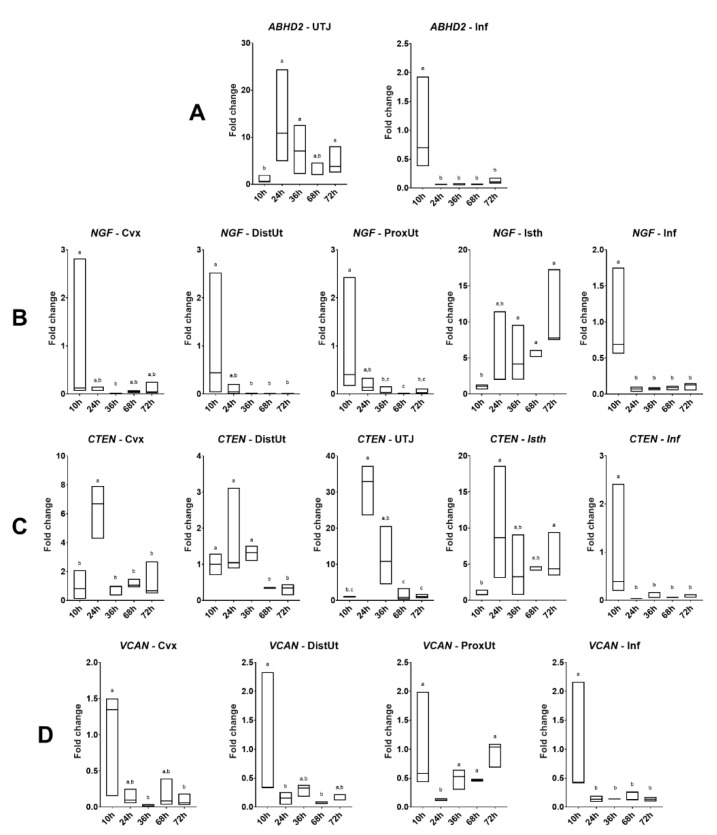
Results of Experiment 2. Gene expression of (**A**) *ABHD2*, (**B**) *NGF*, (**C**) *CTEN*, and (**D**) *VCAN* was statistically significant (*p* < 0.05) in rabbit endocervix (Cvx), distal uterus (DistUt), proximal uterus (ProxUt), utero-tubal junction (UTJ), distal isthmus (Isth), ampulla (Amp), and infundibulum (Inf) 20 h after 10, 24, 36, 68, or 72 h post-induction of the ovulation (intramuscular injection of 0.03 mg gonadotropin-releasing hormone) and natural mating (*n* = 3/collection time). Fold changes relative to 10 h post-mating group are shown. Different letters (^a,b,c^) represent statistical differences between groups (*p* < 0.05). Median [minimum, maximum].

Differences in *ABHD2*, *NGF*, *CTEN*, and *VCAN* expression among groups included in Experiment 2 are displayed in Figure 3. The *ABHD2* expression was upregulated at 24, 36, and 72 h post-mating in the utero-tubal junction (*p* < 0.05), whereas it was downregulated at 24, 36, 68, and 72 h post-mating in the infundibulum (*p* < 0.001). The *NGF* expression was downregulated in the endocervix at 36 h post-mating (*p* < 0.01); downregulated in the distal and proximal uterus at 36, 68, and 72 h post-mating (*p* < 0.05); upregulated in the distal isthmus at 36, 68, and 72 h post-mating (*p* < 0.05); and downregulated in the infundibulum at 24, 36, 68, and 72 h post-mating (*p* < 0.001). The *CTEN* expression followed an *NGF* expression pattern with a time lag of hours in the uterine tissues. At 24 h post-mating, the *CTEN* expression was upregulated in the endocervix (*p* < 0.01). Additionally, the *CTEN* expression was downregulated in the distal uterus at 68 and 72 h post-mating (*p* < 0.05); upregulated in the utero-tubal junction at 24 h post-mating (*p* < 0.001); upregulated in the isthmus at 24 and 72 h post-mating (*p* < 0.05); and downregulated at 24, 36, 68, and 72 h post-mating (*p* < 0.05). The *VCAN* expression was downregulated in the endocervix at 36 and 72 h post-mating (*p* < 0.05); downregulated in the distal uterus at 24 and 68 h post-mating (*p* < 0.05); downregulated at 24 h post-mating in the proximal uterus (*p* < 0.001); and downregulated in the infundibulum at 24, 36, 68, and 72 h post-mating (*p* < 0.01). The results obtained from all of the genes and tissues analyzed are depicted in Figure S2.

### 3.3. ABHD2 and CTEN Follow a Similar Expression Pattern in Tissues at 20 h Post-Mating

**Figure 4:**
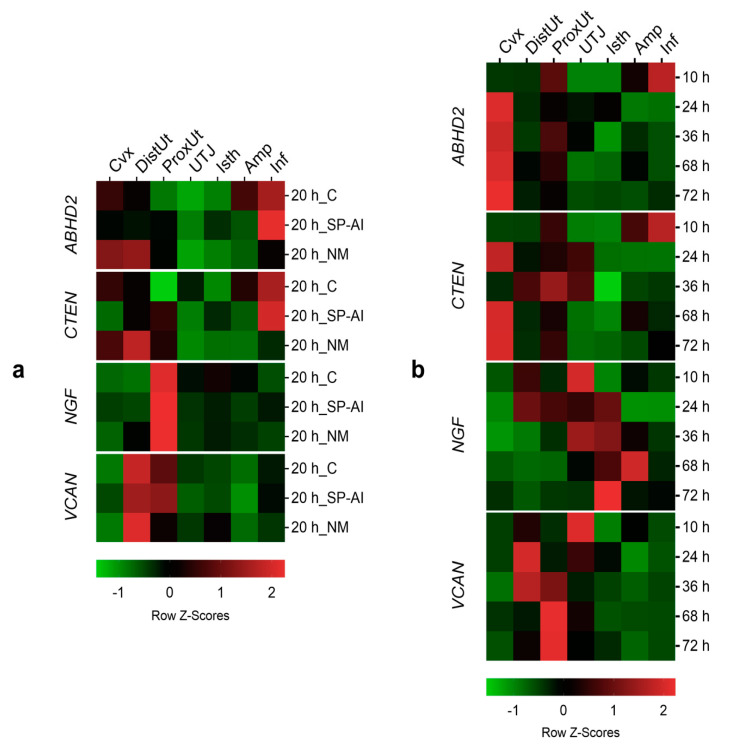
Gene expression changes among tissues in the different groups included in the study. Changes in *ABHD2*, *CTEN*, *NGF*, and *VCAN* expression among different tissues (endocervix, Cvx; distal uterus, DistUt; proximal uterus, ProxUt; utero-tubal junction, UTJ; distal isthmus, Isth; ampulla, Amp; and infundibulum, Inf) (**a**) at 20 h after the induction of ovulation with 0.03 mg of gonadotropin-releasing hormone (GnRH) intramuscularly, as the control of ovulation (20 h_C); 20 h post-GnRH-stimulation and sperm-free seminal plasma vaginal infusion (20 h_SP-AI); and 20 h post-GnRH-stimulation and natural mating (20 h_NM); (**b**) at 10, 24, 36, 68, and 72 h post-GnRH-stimulation and natural mating. Row Z-Scores of the mean fold change relative to the reference group (UTJ) are shown. Red indicates upregulation and green indicates downregulation.

The tissue analysis expression showed significant differences in *ABHD2*, *CTEN*, *NGF*, and *VCAN* expression among tissues in the different groups included in Experiment 1 (Figure 4a, Figure S3). The *ABHD2* and *CTEN* expression presented a similar tissue pattern. The infundibulum presented the highest *ABHD2* and *CTEN* expression in the SP-AI and control groups (*p* < 0.05), whereas the distal uterus, followed by the endocervix, presented the highest *ABHD2* and *CTEN* expression in the NM group (*p* < 0.01). In the case of *NGF* expression, the proximal uterus presented the highest *NGF* expression in all groups included in Experiment 1 (*p* < 0.01). The highest *VCAN* expression in the control and NM groups was present in the distal uterus (followed by the proximal uterus in the control group) (*p* < 0.05), whereas the proximal uterus, followed by the distal uterus, presented the highest *VCAN* expression in the SP-AI group (*p* < 0.01).

### 3.4. NGF Follows an Up-Reguation Wave Expression Pattern in a Post-Mating Time-Dependent Manner

The tissue analysis expression showed significant differences in *ABHD2*, *CTEN*, *NGF*, and *VCAN* expression among tissues in the different groups included in Experiment 1 (Figure 4b, Figure S4).

As seen in the tissue expression analysis of Experiment 1 (Figure 4a), *ABHD2* and *CTEN* expressions followed a similar tissue pattern in Experiment 2. At 10 h post-mating (time of ovulation), both genes were upregulated in the infundibulum (*p* < 0.05), whereas for the remaining times, post-mating presented higher expressions in the uterine tissues (endocervix, distal uterus, and proximal uterus) (*p* < 0.05). The *NGF* expression in the different tissues seemed to follow an upregulated wave along the oviduct tissues beginning, with the maximum Z-score, at 36 h post-mating in the utero-tubal junction and distal isthmus (*p* < 0.05), and finishing in the distal isthmus at 72 h post-mating (*p* < 0.01), moving through the ampulla at 68 h post-mating (*p* < 0.05). Finally, the uterine tissues (distal and proximal uterus) presented the highest *VCAN* expression at 36, 68, ad 72 h post-mating (*p* < 0.05).

### 3.5. Ovulated Follicles and Embryo Recovery

The number of ovulated ovarian follicles, and the number and stage of the embryos collected were counted for each doe (Table 2).

## 4. Discussion

In this study, we investigated the differential expression of four genes (*ABHD2*, *VCAN*, *NGF*, and *CTEN*) associated with sperm-lining epithelium interactions (adhesion), ovulation, and pre-implantation effects (which can be separated from the un-implanted embryo influence) in the period 10–72 h post-mating, during which all of these events occur. Our study provides the basis for new mechanistic approaches that can lead to novel strategies for improving fertility in rabbit production.

Two independent types of statistical analyses were included in the present study to emphasize differences between groups and tissues. The first statistical analyses compared experimental groups in the same tissue segments, whereas the second statistical analyses compared tissue segments in the same experimental groups. The results can vary and appear to disagree in some cases because the information extracted from each analysis is complementary, not the same.

The oviduct is involved in the transport of spermatozoa and oocytes to the site of fertilization, as well as the maintenance of sperm viability [7,16]. This interaction preserves the spermatozoa in a storage reservoir until ovulation and maintains their fertilization competence [8]. Our analyses revealed no effect on the gene expression of the selected genes by mating or sperm-free SP in the sperm reservoir at 20 h post-exposure. These results suggest that, even though the role of the sperm reservoir is fundamental during the first 2–3 h post-ovulation, the expression of the selected genes was not affected by natural mating or sperm-free SP vaginal infusion after 10 h post-ovulation. However, adjacent tissues of the sperm reservoir, such as the utero-tubal junction, presented differential *ABHD2* expression at 20 h post-exposure.

According to the principle of the oviductal sperm reservoir explained above, we hypothesized that the *ABHD2* expression should decrease in the distal isthmus before ovulation, repressing, in this way, sperm capacitation and, just after ovulation (10 h post-mating), the *ABHD2* expression should increase to facilitate the detachment of the oviductal sperm reservoir and spermatozoa-oocyte contact. However, our observations found no differences in *ABHD2* expression in the distal isthmus at different hours post-mating up to 72 h. Despite the lack of differences in the distal isthmus, at 20 h post-mating, the *ABHD2* expression was downregulated in the utero-tubal junction. These findings suggest that the utero-tubal junction instead of the distal isthmus, or the utero-tubal junction in conjunction with the distal isthmus, may be the anatomical region of the doe reproductive tract that acts as a potential sperm reservoir in rabbits. Additionally, the *ABHD2* expression decreased in the utero-tubal junction at the time of ovulation, increasing around 24 h post-mating until 72 h post-mating. Our observations may be relevant for understanding events during sperm capacitation, as well as for early embryo development. However, further research is needed to clarify the exact mechanism of action of the *ABHD2* in the oviductal segments of the rabbit.

The analysis of *VCAN* expression revealed no effect of natural mating and sperm-free SP at 20 h post-exposure. In contrast, expression values from 10 to 72 h post-mating showed a dual regulation of *VCAN* expression. On one hand, the data suggest a probable implication of the oviductal extracellular matrix in the oocyte picked up by the infundibulum close to expected ovulation, showing the downregulation of *VCAN* expression at different times post-mating compared to 10 h post-mating. On the other hand, the endometrial extracellular matrix could be involved in the entrapment of spermatozoa in proximal and distal parts of the uterus and endocervix at the time of ovulation (10 h post-mating), in order to prevent the forward motion of spermatozoa to the utero-tubal junction and the oviduct. This hypothesis is in accordance with previous research proposing the cervix as the first anatomical barrier for spermatozoa progression in rabbits, from which viable spermatozoa progress to the oviduct into the site of fertilization [6,35,36]. Other anatomical structures have been proposed as barriers to sperm progression [35], but it seems that the distal isthmus acts as the oviductal sperm reservoir in rabbits [10].

Versican also plays a fundamental role in embryo implantation [28], suggesting that its expression should be increased in the endometrium before implantation takes place. Our study revealed an early upregulation of *VCAN* expression in the distal uterus in all groups at 20 h post-exposure. This upregulation remained from 36 h until 72 h post-mating in the proximal uterus, indicating that an appropriate environment for embryo implantation is occurs a few hours after ovulation and before the embryos reach the endometrium.

Recent studies have identified the NGF as an ovulatory induction factor present in the female and male genital tract in some reflex ovulating species, such as camelids and rabbits [24,37,38,39]. The biological actions of NGF are mediated by two neurotrophin receptors: the tropomyosin receptor kinase A (TrkA, specific for NGF) and the p75 neurotrophin receptor (p75-NTR, a pan-neurotrophin receptor with a low affinity to NGF) [40]. Both receptors are present in male rabbits [21,22,23,39] and female reproductive tracts [24,41]. Two complementary mechanisms for the induction of ovulation in rabbits via NGF have been proposed [24]. One of the mechanisms is based on an endocrine ovulation-inducing factor-mediated pathway, where NGF is mainly synthesized in the uterine wall and absorbed into the bloodstream and directly acts on the ovary [24]. At the same time, a complementary nervous NGF-mediated pathway could be produced by the direct action of NGF on primary sensory neurons which trigger GnRH neurons in the hypothalamus [24].

Our results revealed an upregulation of *NGF* expression at 20 h post-mating in the distal and proximal uterus, suggesting that mating, but not sperm-free SP infusion, is involved in the *NGF* regulation on uterine tissues. In relation to this effect, the endocervix, the proximal and distal uterus, and the infundibulum followed a similar uterine tissue pattern upregulating *NGF* at 10 h post-mating. Taken together, endocervix and endometrial tissues, in conjunction with the infundibulum, may act as the main sources of NGF based on the endocrine ovulation-inducing factor-mediated pathway [24].

Once ovulation occurs, the unfertilized oocytes migrate along half of the total length of the oviduct within the first 2 h post-ovulation [42]. At the same time (2–3 h post-ovulation), fertilization takes place near the ampulla [10,15,35]. Embryos are delayed at the ampulla-isthmus junction until 48 h post-mating and then pass through the isthmus portion after 70 h post-mating to finally pass through the utero-tubal junction and enter the uterus [42]. The analyses of *NGF* expression showed an upregulation in the isthmus at 36, 68, and 72 h post-mating, suggesting that embryos are naturally exposed to NGF during its passage along the isthmus before implantation, probably to enhance embryo hatching rates in a similar way as reported previously in a rabbit in vitro embryo development study [43]. However, the mechanisms by which NGF exerts such actions are still unclear.

It is hypothesized that NGF may assist in embryo implantation through the inhibition of the local immune response [20] and is able to enhance endogenous anti-inflammatory mechanisms on monocytes via the activation of TrkA signaling, inhibiting the production of proinflammatory cytokine, including the interleukins (IL) IL-1β and IL-6 and tumor necrosis factor α (TNF-α), and increasing the production of the anti-inflammatory cytokines IL-10 and IL-1Ra [44]. Our study included an interval up to 72 h post-mating. Therefore, it only included preimplantation stages. However, the upregulation of *NGF* expression observed in the isthmus at 36, 68, and 72 h post-mating and the wave of *NGF* expression that began at 36 h post-mating in the utero-tubal junction and finished in the isthmus at 72 h post-mating, moving through the ampulla at 68 h post-mating, may be related to the tolerance to embryos by the immune system of the doe. Moreover, according to the tissue expression analyses, the presence of *NGF* expression in the proximal uterus 10 h after ovulation (20 h post-mating) may be related to reducing the inflammatory response produced by ovulation and mating in the reproductive tract.

The NGF can upregulate the expression of CTEN, a distant member of the tensin focal adhesion family, mainly via RAS-Raf-Mek, PI3K-Akt, and Stat3 pathways [45]. The CTEN is involved in cell motility, apoptosis, growth factor receptor homeostasis, and tumorigenicity [30]. Its expression shows a restricted pattern in the human prostate and placenta [46]. In this study, we have demonstrated its expression in the doe reproductive tract. Recent research has described its differential expression in the pig female reproductive tract induced by spermatozoa and sperm-free SP [19], indicating that CTEN might be related to an early signaling mechanism of the female reproductive tract in response to both spermatozoa and SP.

Our results suggest that mating, but not sperm-free SP, is involved in *CTEN* upregulation in the proximal and distal uterus. Similarly, the *CTEN* expression was upregulated at 20 h post-mating in the endocervix, suggesting that mating is an effector of cell migration and the apoptosis response against spermatozoa in the cervix and uterus (proximal and distal), probably related to a reduction of the spermatozoa population in the uterine lumen, as previously reported [6,35]. Furthermore, the results suggest that *CTEN* expression follows a similar *NGF* expression pattern in the uterus (proximal and distal) when mating is involved, whereas *CTEN* expression follows a similar *NGF* expression pattern in the endocervix when sperm-free SP infusion is involved. Interestingly, our analyses showed a similar expression, of *NGF* and *CTEN*, both upregulated, at 10 h post-mating compared with the remaining times post-mating. Accordingly, *CTEN* expression followed a similar *NGF* expression, with a time lag of hours in some cases, in the different post-mating times in the endocervix, distal uterus, and distal isthmus. However, the exact mechanism of this interaction is unknown. In a similar way to *ABHD2* expression, the tissue expression analyses conducted 20 h after treatment demonstrated differential *CTEN* expression that switches from infundibulum to uterine tissues, but only if fertilization takes place. The specific mechanisms involved in this differential expression in the female reproductive tract are unknown and require further investigation.

The mid-ampulla has been proposed as the site of fertilization in rabbits [10,15,35]. Curiously, no effects on *VCAN*, *ABHD2*, *NGF*, and *CTEN* expression were found in the ampulla through time. It should be noted that a lack of gene expression changes in the ampulla is not unexpected given the fast passage of the oocytes through this anatomical region, with them spending more time in the ampulla-isthmus junction during isthmic migration [42], which is the anatomical region where we found numerically more gene expression variations among the different conditions and times included.

## 5. Conclusions

In conclusion, our findings suggest that the endocervix and the uterine horns, in conjunction with the infundibulum, are involved in the endocrine ovulation-inducing factor NGF-mediated pathway. Additionally, we propose the implication of *NGF* in the maternal environment-embryo interaction, perhaps controlling inflammation of the reproductive tract, and embryo survival. However, the mechanisms by which *NGF* exerts such actions are unknown. Furthermore, we reported the differential expression of other genes (*ABHD2*, *CTEN*, and *VCAN*) involved in the endometrium-sperm interaction and embryo implantation. The findings of the present study should form the basis of new mechanistic approaches for developing new strategies for the improvement of fertility in animal production.

## Figures and Tables

**Table 1:** Primers used for the quantitative PCR analyses.

Gene	Primer Sequence (5′–3′)	Product Size (bp)	Efficiency (%)
*ABHD2*	F: CGGAGCCACTTCTACTTTCG	159	87.7
	R: GCACACCGATAGCCATTTTT		
*β-ACTIN*	F: unknown	120	88.6
	R: unknown		
*CTEN*	F: TGGTCCACTTCAGAGTCACG	211	97.3
	R: AAGAGGTGGCACACGTTCTC		
*NGF*	F: CCCCTCCAACAGGACTTACA	138	103.7
	R: ACCTCATTGCCCTTGATGTC		
*VCAN*	F: TGCACCACAACCAACAGATT	177	89.5
	R: AGCTGCGAAGAGATGTGGTT		

*ABHD2*: α/β hydrolase domain-containing protein 2; *β-ACTIN*: β-actin; *CTEN*: C-terminal tensin-like protein; *NGF*: nerve growth factor; *VCAN*: versican; F: forward; R: reverse; A: adenine; C: cytosine; G: guanine; T: thymine; bp: base pair.

**Table 2:** Number of ovulated follicles and embryo recovery from both reproductive tract sides of the does included in this study.

Time Post-Mating	Ovulated Follicles	Embryo Recovery	Embryo Stage
24 h	6.17 ± 0.90	3.17 ± 2.41	2 and 4-cell
36 h	4.67 ± 2.81	3.67 ± 2.81	8-cell
68 h	5.67 ± 2.21	5.67 ± 1.80	Early morula
72 h	4.00 ± 0.58	4.17 ± 0.37	Morula

Data are presented as the mean ± SD.

## Supplementary Materials

The following are available online at https://www.mdpi.com/2073-4425/11/7/758/s1: Figure S1. Changes in (A) ABHD2, (B) CTEN, (C) NGF, and (D) VCAN expression among different treatments at 20 h post-treatment: 20 h post-induction of ovulation, control (20 h_C); 20 h post-seminal plasma infusion, 20 h_SP; and 20 h post-natural mating, 20 h_NM. Tissue anatomical regions of the female rabbit reproductive tract (endocervix, Cvx; distal uterus, DistUt; proximal uterus, ProxUt; utero-tubal junction, UTJ; distal isthmus, Isth; ampulla, Amp; and infundibulum, Inf). Fold changes relative to the reference group (20 h_C) are shown. Different letters (a,b) represent statistical differences between tissues (*p* < 0.05). Median [minimum, maximum]; Figure S2. Changes in (A) ABHD2, B) CTEN, C) NGF, and D) VCAN expression at different times (from 10 to 72 h) post-mating: 10, 24, 36, 68, and 72 h post-natural mating. Tissue anatomical regions of the female rabbit reproductive tract (endocervix, Cvx; distal uterus, DistUt; proximal uterus, ProxUt; utero-tubal junction, UTJ; distal isthmus, Isth; ampulla, Amp; and infundibulum, Inf). Fold changes relative to the reference group (10 h post-mating) are shown. Different letters (a–c) represent statistical differences between tissues (*p* < 0.05). Median [minimum, maximum]; Figure S3. Changes in (A) ABHD2, (B) CTEN, (C) NGF, and (D) VCAN expression among different tissues at 20 h post-treatment. 20 h post-induction of the ovulation, control (20 h_C); 20 h post-seminal plasma infusion, 20 h_SP; and 20 h post-natural mating, 20 h_NM. Tissue anatomical regions of the female rabbit reproductive tract (endocervix, Cvx; distal uterus, DistUt; proximal uterus, ProxUt; utero-tubal junction, UTJ; distal isthmus, Isth; ampulla, Amp; and infundibulum, Inf). Fold changes relative to the reference group (UTJ) are shown. Different letters (a–d) represent statistical differences between tissues (*p* < 0.05). Median [minimum, maximum]; Figure S4. Changes in (A) ABHD2, (B) CTEN, (C) NGF, and (D) VCAN expression among different tissues in the period 10–72 h (10, 24, 36, 68, and 72 h post-natural mating). Tissue anatomical regions of the female rabbit reproductive tract (endocervix, Cvx; distal uterus, DistUt; proximal uterus, ProxUt; utero-tubal junction, UTJ; distal isthmus, Isth; ampulla, Amp; and infundibulum, Inf). Fold changes relative to the reference group (UTJ) are shown. Different letters (a–d) represent statistical differences between tissues (*p* < 0.05). Median [minimum, maximum].
