# Supplementary material for: Semen Modulates the Expression of NGF, ABHD2, VCAN, and CTEN in the Reproductive Tract of Female Rabbits
Source: Genes (Basel). 2020 Jul 7;11(7):758. doi: 10.3390/genes11070758 (PMC7397043; doi:10.3390/genes11070758)
Supplement: Supplementary file 1 [file genes-11-00758-s001.pdf]

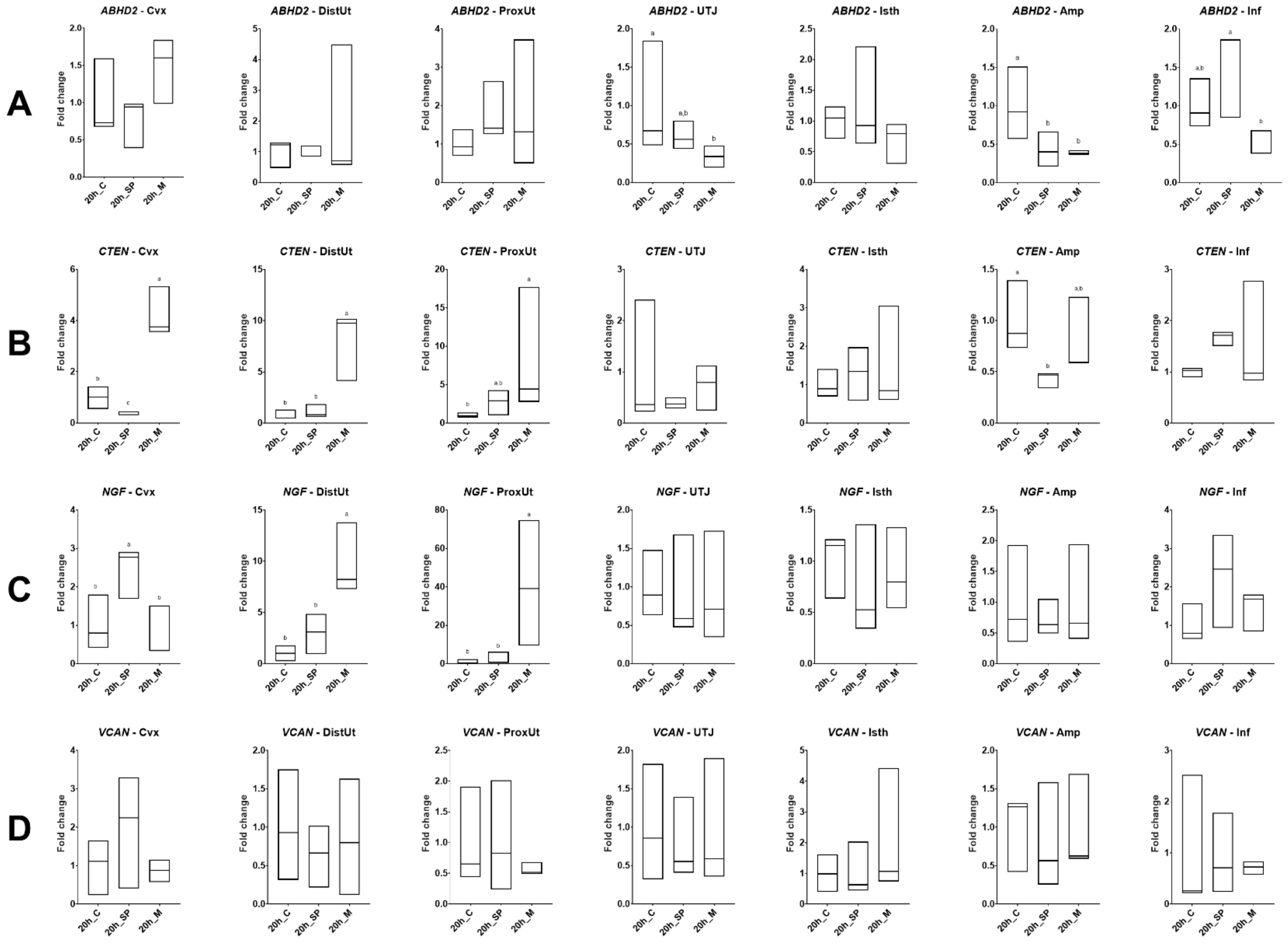

Figure S1. Changes in **A**) *ABHD2*, **B**) *CTEN*, **C**) *NGF* and **D**) *VCAN* expression among different treatments at 20 h post-treatment: 20 h post-induction of the ovulation, control (20 h_C); 20 h post-seminal plasma infusion, 20 h_SP; and 20 h post-natural mating, 20 h_NM. Tissue anatomical regions of the rabbit female reproductive tract (endocervix, Cvx; distal uterus, DistUt; proximal uterus, ProxUt; utero-tubal junction, UTJ; distal isthmus, Isth; ampulla, Amp; and infundibulum, Inf). Fold changes relative to reference group (20 h_C) are shown. Different letters (^{a,b}) represent statistical differences between tissues ($p < 0.05$). Median [minimum, maximum].

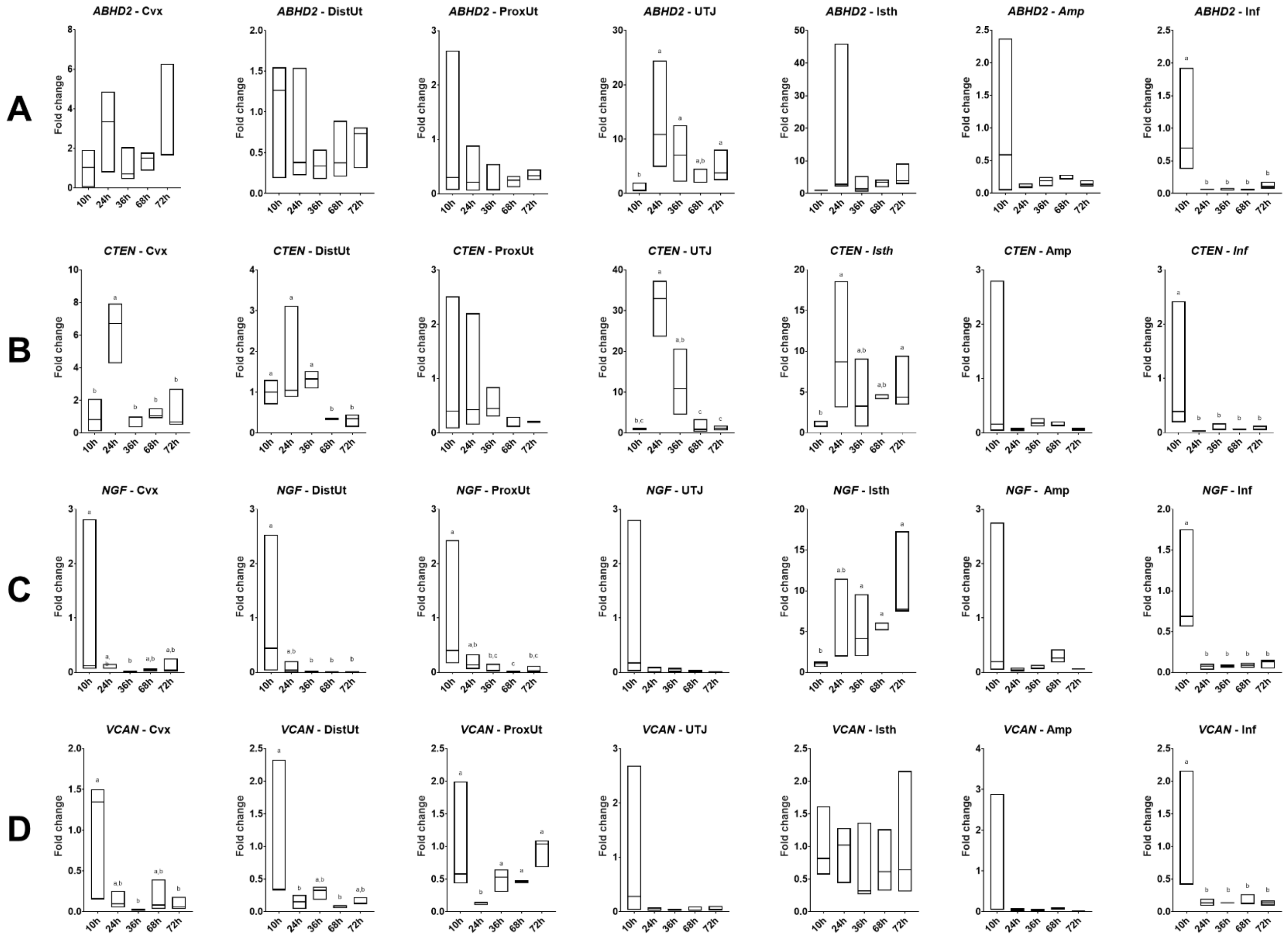

Figure S2. Changes in **A**) *ABHD2*, **B**) *CTEN*, **C**) *NGF*, and **D**) *VCAN* expression among different times (from 10 to 72 h) post-mating: 10, 24, 36, 68, and 72 h post-natural mating. Tissue anatomical regions of the rabbit female reproductive tract (endocervix, Cvx; distal uterus, DistUt; proximal uterus, ProxUt; utero-tubal junction, UTJ; distal isthmus, Isth; ampulla, Amp; and infundibulum, Inf). Fold changes relative to reference group (10 h post-mating) are shown. Different letters (^{a,b,c}) represent statistical differences between tissues ($p < 0.05$). Median [minimum, maximum].

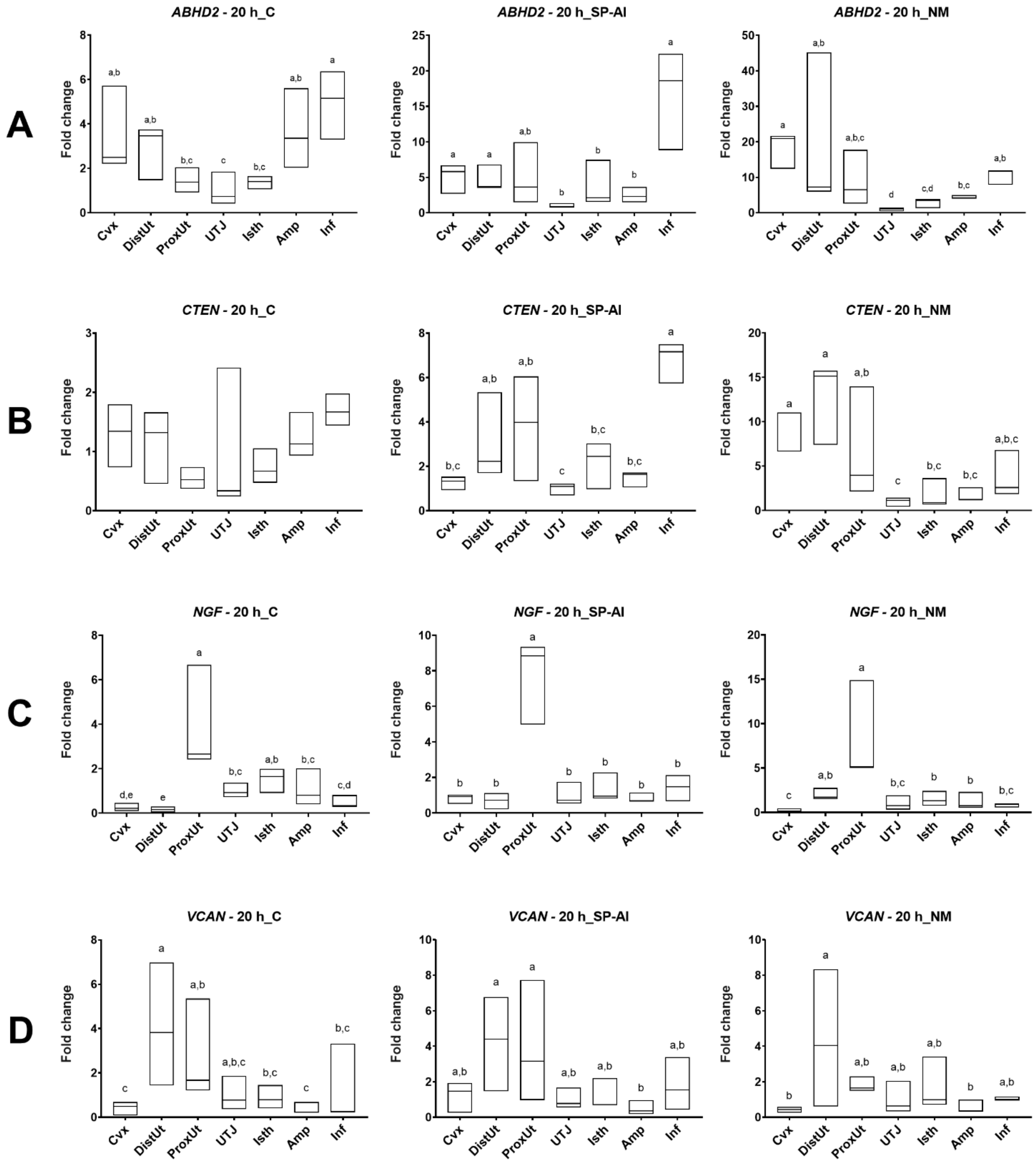

Figure S3. Changes in **A**) *ABHD2*, **B**) *CTEN*, **C**) *NGF*, and **D**) *VCAN* expression among different tissues at 20 h post-treatment. 20 h post-induction of the ovulation, control (20 h_C); 20 h post-seminal plasma infusion, 20 h_SP; and 20 h post-natural mating, 20 h_NM. Tissue anatomical regions of the rabbit female reproductive tract (endocervix, Cvx; distal uterus, DistUt; proximal uterus, ProxUt; utero-tubal junction, UTJ; distal isthmus, Isth; ampulla, Amp; and infundibulum, Inf). Fold changes relative to reference group (UTJ) are shown. Different letters (^{a,b,c,d}) represent statistical differences between tissues ($p < 0.05$). Median [minimum, maximum].

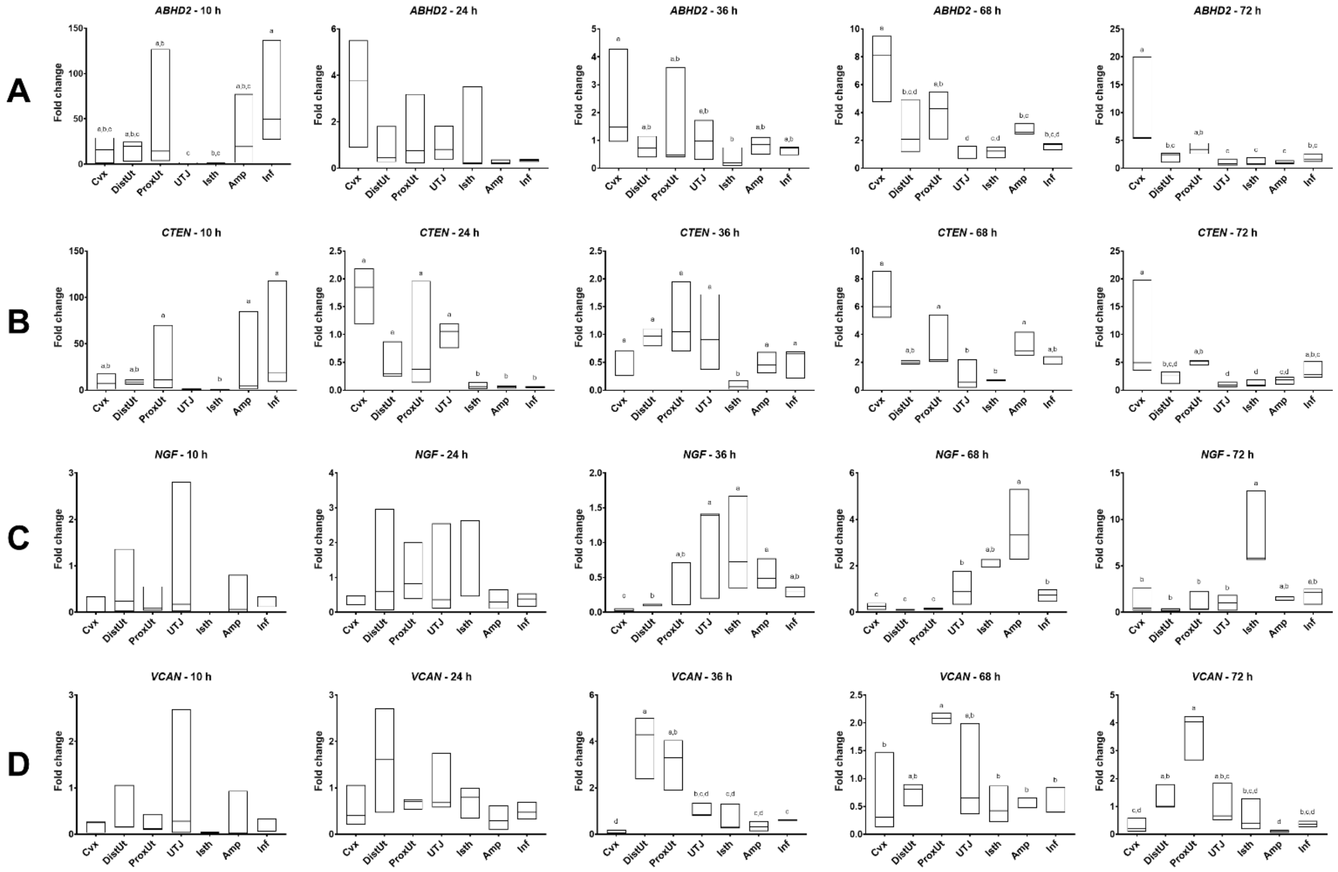

Figure S4. Changes in **A**) *ABHD2*, **B**) *CTEN*, **C**) *NGF*, and **D**) *VCAN* expression among different tissues in the period 10-72 h. 10, 24, 36, 68, and 72 h post-natural mating. Tissue anatomical regions of the rabbit female reproductive tract (endocervix, Cvx; distal uterus, DistUt; proximal uterus, ProxUt; utero-tubal junction, UTJ; distal isthmus, Isth; ampulla, Amp; and infundibulum, Inf). Fold changes relative to reference group (UTJ) are shown. Different letters (^{a,b,c,d}) represent statistical differences between tissues ($p < 0.05$). Median [minimum, maximum].
